# Prognostic predictors of mortality among individuals with advanced dementia residing in nursing homes: a prospective cohort study

**DOI:** 10.1186/s12877-025-06350-5

**Published:** 2025-10-22

**Authors:** Jinfeng Wang, Xiaoling Wang, Yan Zhuo, Jirong Yue, Birong Dong, Xue Yang, Ying Li

**Affiliations:** 1 The Center of Gerontology and Geriatrics, National Clinical Research Center for Geriatrics, West China Hospital, Sichuan University, Sichuan, China; 2 West China Hospital, Sichuan University, Sichuan, China

**Keywords:** Advanced dementia, Palliative care, Mortality, Prognostic factors, Memantine

## Abstract

**Background:**

Limited research has explored survival outcome predictions in those with advanced dementia within the Chinese population. A longitudinal cohort study was performed to better characterize the end-of-life trajectories of Chinese individuals with advanced dementia, providing a means of identifying prognostic predictors of 32-month mortality among nursing home residents in Chengdu, China.

**Methods:**

This study enrolled 186 residents who met the inclusion criteria, with all having advanced dementia. Participants were evaluated at baseline and followed until death or the study’s conclusion. Demographic information, health and medical status, and mortality-related data were assessed. Kaplan-Meier survival analyses and Cox proportional hazards models were applied to approximate survival rates and identify predictive factors.

**Results:**

The median age of enrolled participants was 83.5 years, with females comprising 60.2% of the cohort. The overall survival rate was 52.0%. After adjusting for all variables, three significant prognostic factors were identified: oral nutritional supplement use (HR: 3.006, 95% CI: 1.593–5.671, *p* = 0.001), albumin concentration (HR: 0.914, 95% CI: 0.861–0.970, *p* = 0.003), and memantine use (HR: 0.416, 95% CI: 0.190–0.907, *p* = 0.028). Survival at 6 months, 1 year, and 2 years was significantly higher in the memantine group versus non-users.

**Conclusions:**

The present study identified key prognostic factors for mortality in Chinese nursing home residents with advanced dementia. Notably, memantine use emerged as a protective factor, marking the first demonstration of its potential survival benefit in this population. These findings may aid healthcare professionals in optimizing resource allocation and care strategies for individuals at the end of life.

**Trial registration:**

ChiCTR2100041764 (Chinese Clinical Trial Registry). Registered 4 January 2021.

**Supplementary Information:**

The online version contains supplementary material available at 10.1186/s12877-025-06350-5.

## Introduction

Dementia is a progressive neurological disorder marked by continuous deterioration in cognitive function, which gradually impairs daily functioning and undermines independence. As the disease advances, affected individuals require increasingly complex care, imposing significant emotional, physical, and economic burdens on families, caregivers, and society at large [1]. The 2021 Global Burden of Disease (GBD) study reported that the impact of Alzheimer’s disease and other dementias (ADOD) in Asia has risen sharply between 1990 and 2021. During this period, the prevalence, incidence, mortality, and disability-adjusted life years (DALYs) of ADOD increased by 250.44, 244.73, 297.34, and 249.54%, respectively. Projections suggest that the burden of ADOD will continue to grow over the next three decades, underscoring an urgent need for enhanced public health strategies and care infrastructure across the region [2]. Dementia ranks among the primary causes of illness and death globally and is associated with greater mortality risk. The disease follows a highly variable clinical trajectory, with a median survival after diagnosis of between 3.3 and 11.7 years. Most of this time is spent in the most severe stage of the disease [3].

Advanced dementia, the final stage of the disease characterized by profound cognitive impairment, severe functional decline, and notable behavioral changes [4]. This stage is associated with high mortality. In the Choices, Attitudes, and Strategies for Care of Advanced Dementia at the End-of-Life (CASCADE) study [5], which studied nursing home residents and entailed an 18-month evaluation of 323 residents with advanced dementia, a median survival period of 1.3 years was noted. Frequently reported complications included eating difficulties (86%), episodes of fever (53%), and pneumonia (41%). The primary goal of treatment is to extend survival while maintaining quality of life; however, there remain significant difficulties in predicting life expectancy among advanced dementia patients.

Various studies have explored various factors linked to mortality in individuals with dementia, including biomarkers like homocysteine, clinical indices such as the Modified Shock Index, and measures of cardiorespiratory fitness [6–8]. However, research specifically addressing mortality predictors in advanced dementia particularly within nursing home populations remains scarce and is largely based on Western cohorts. In an investigation by Kathryn L. Hicks et al., the roles of medical comorbidities such as psychosis, pulmonary disease, diabetes, pneumonia, and heart conditions wer'e investigated concerning survival outcomes. Among these, pneumonia was the only comorbidity that was significantly linked with reduced survival in cases of advanced dementia [9]. Similarly, a prospective cohort study by Daichi Hoshino et al. reported associations between masseter muscle-tension palpation and modified water-swallowing tests with one-year mortality of advanced dementia [10]. Several prognostic models, such as hospice eligibility guidelines and the Advanced Dementia Prognostic Tool (ADEPT), have been developed to approximate 6-month survival among those with advanced dementia who are residing in nursing homes [11, 12]. A majority of other studies assessing hospice eligibility guidelines and ADEPT have centered on Caucasian populations, including data derived from Western nations and the associated healthcare systems among the data used to evaluate residents in nursing homes and conduct care-related screening efforts [13]. Based on the most representative study, a mean ADEPT score of 10.1 is linked to a 6-month mortality rate of 18.3% in North America [11]. In contrast, there is a lack of research focused on predicting survival outcomes among advanced dementia targeting Chinese people. A study by Junjin Liu et al. investigated advanced dementia survival in Chongqing nursing homes, reporting a 6-month mortality rate of only 4.3%, despite a mean ADEPT score of 13.0 [14]. This discrepancy suggests that ADEPT may not be an ideal prognostic tool for Chinese nursing home residents. Notably, while prior pharmacological studies in dementia populations primarily examined impacts on cognitive function, neuropsychiatric symptoms, and physical functioning [15–17], few studies have explored the relationship between specific medication use, such as anti-dementia agents, sedative-hypnotics, anti-psychotics, and anti-depressants, and mortality in this advanced-stage population. To address this knowledge gap, a longitudinal prospective cohort study was herein performed to identify key prognostic predictors of 32-month mortality among nursing home residents with advanced dementia in Chengdu, China.

## Methods

### Study design and setting

This prospective observational cohort study was performed across three nursing homes in Chengdu, China. Participant recruitment took place between January 1 and August 30, 2021. Detailed medical information was systematically collected, including electronic health records, past medical history, and laboratory results. Residents were monitored until death or the study endpoint on September 30, 2023. The primary outcomes assessed were all-cause mortality and overall survival duration.

### Study population

The study was conducted in accordance with the Declaration of Helsinki and approved by the Ethics Review Committee of The West China Hospital of Sichuan University (Approval No. 2020 − 1255). For adults with advanced dementia who lacked decision-making capacity, assent was gained from the next-of-kin or the agreed family carer(s) of the participants. All next-of-kin or the agreed family carer(s) of participants were then thoroughly informed about the purpose of the study and provided written informed consent prior to participation. If there was no family carer(s) available, the research team sought assent from the agreed healthcare professional/healthcare institution responsible for making decisions for the participants.

Although no universally accepted definition for advanced dementia exists, previous research has characterized its key clinical features as including severe memory impairment (such as failure to recognize close family members), significantly limited verbal communication, loss of mobility, complete dependence in daily living activities, and both urinary and fecal incontinence [5]. These characteristics align with Global Deterioration Scale (GDS) stages 6 and 7, indicating severe cognitive deterioration, respectively. In their study on prognostic tools for survival prediction in advanced dementia, Chinese researchers Junjin Liu and colleagues classified individuals at GDS stage 6 or higher as having advanced dementia [14]. Accordingly, informed by existing clinical guidelines, research literature, and diagnostic frameworks, advanced dementia in this investigation was classified according to GDS stage 6 or higher.

Inclusion criteria were: age ≥ 65 years, a dementia diagnosis (any type) confirmed in medical records, and GDS stage of 6 or higher. Residents in a biologically terminal state (clinically unstable with an irreversible risk of death) or those with advanced cancer were excluded. Based on these criteria, 186 residents with advanced dementia were enrolled for follow-up observation.

### Data collection

Demographic characteristics, including age, gender, and nursing home location, were recorded. The GDS, developed by Reisberg et al., was employed to evaluate dementia severity [18]. Although an official Chinese version of the GDS has not yet been published, this study used a translated version. While the reliability and validity of this translation have not been formally specified, the tool has been increasingly adopted in research involving Chinese populations. For instance, a randomized, open-label pilot study assessing the influence of topical emollients on cognitive function in older individuals used the GDS to evaluate dementia severity. A marked rise in the GDS scores from baseline was seen in the controls (*P* < 0.001), whereas GDS scores remained stable in those receiving treatment [19]. Another cohort study investigating prognostic factors for neurological outcomes in children following carbon monoxide poisoning employed the GDS to differentiate between those with and without cognitive impairment [20]. The GDS evaluates several domains, including memory, instrumental activities of daily living (IADLs), changes in personality and mood, basic activities of daily living (ADLs), and orientation, among others. It categorizes cognitive deterioration into seven stages, namely, none, very mild, mild, moderate, moderately severe, severe, and very severe cognitive decline. Detailed assessment criteria for each stage are provided in Table S1 of Additional File 1 [18].

The Quality of Life in Late-Stage Dementia (QUALID) Scale, created by Weiner MF and colleagues, is a caregiver-reported tool specifically developed to evaluate the quality of life in individuals with advanced dementia [21]. Mao Pan and colleagues translated the scale into Chinese and assessed its suitability in China, demonstrating strong reliability and validity [22]. The 11-item scale assesses behavioral and emotional indicators observed by caregivers in individuals with late-stage dementia over the preceding week. Items are scored on a 5-point scale, resulting in an overall score between 11 and 55, where lower scores are indicative of higher quality of life (detailed scoring criteria for each item are provided in Table S2, Additional File 1).

The Bedford Alzheimer Nursing Severity Scale (BANS-S), developed by Volicer et al., is a validated tool that evaluates disease severity in individuals with late-stage Alzheimer’s dementia. In this study, we applied a translated version of the scale. The BANS-S integrates assessments of cognitive and functional impairments and common clinical symptoms, and includes seven polytomous items: two assessing cognitive functions (speech and eye contact), three evaluating functional abilities (dressing, eating, and ambulation), and two addressing pathological symptoms (disturbances in the sleep–wake cycle and muscle rigidity or contractures). Items are scored from 1 to 4, yielding an overall score between 7 and 28 (detailed item criteria are provided in Table S3, Additional File 1) [23].

Nutritional status was evaluated based on left calf circumference, hemoglobin levels, and serum albumin concentration. Participants were categorized into three groups according to their nutritional intake status: Group 1 received oral nutritional supplements (ONS), Group 2 received intravenous nutritional supplements, and Group 3 maintained a regular diet without supplemental nutrition.

Comorbidity data were extracted from the nursing home’s electronic records and included information pertaining to cerebrovascular disease, hypertension, chronic obstructive pulmonary disease (COPD), angina pectoris, connective tissue disease, congestive heart failure, diabetes, peptic ulcers, mild liver disease, moderate to severe renal disease, and hemiplegia.

Acute medical events, such as pneumonia, urinary tract infections (UTI), and fractures occurring within three months before enrollment, were also documented. Additionally, medical interventions administered during the same period were recorded, including oxygen therapy, physical restraint (e.g., bed rails or other immobilizers), antibiotic use, electrocardiogram (ECG) monitoring, gastric tube placement, and urinary catheterization. Medication use was also tracked, focusing on anti-dementia drugs (cholinesterase inhibitors and memantine), sedative-hypnotics (non-benzodiazepine hypnotics, benzodiazepines, and others), anti-psychotic drugs (quetiapine, olanzapine, risperidone, and others), and anti-depressant drugs (citalopram, mirtazapine, sertraline hydrochloride, and fluoxetine hydrochloride).

### Statistical analysis

All statistical analyses, except for the assessment of the proportional hazards assumption, were performed using SPSS for Windows version 29.0, with statistical significance set at a two-tailed *p* < 0.05. Differences between the two groups were analyzed using either the independent samples t-test or the Mann-Whitney U test for continuous variables, which were presented as mean (standard deviation SD) or median (interquartile range IQR). Categorical variables were compared using the chi-square test, with results presented as n (%). Survival time represented the number of months from the enrollment date to the date of death during follow-up, the date the participant was lost to follow-up, or the study endpoint (September 30, 2023), whichever occurred first. A Kaplan-Meier survival model was used to estimate survival time. Participants who were discharged from the nursing home before the end of the follow-up period were considered censored cases. Cox proportional hazards regression models were employed for survival analysis, with results reported as hazard ratios (HRs) alongside 95% Confidence intervals (CIs) and *p*-values. The proportional hazards assumption was evaluated using Schoenfeld residual global and covariate-specific tests implemented via the cox.zph() function in R (version 4.4.1). Graphical diagnostics, including plots of Schoenfeld residuals against time, further supported this assessment. Sensitivity analysis was conducted by re-evaluating the associations between nutritional intake status, memantine use, and mortality after excluding participants who were discharged before the study’s endpoint.

## Results

Overall, 186 residents were enrolled in the present study, of whom 12 (6.4%) were discharged during follow-up, and 85 (45.7%) had died by the study’s endpoint. These participants were a median of 83.5 years old (IQR: 78.0–89.0), with females comprising 60.2% of the sample. All residents had advanced dementia, with 57% classified as GDS stage 7. The most common comorbidities were cerebrovascular disease (76.3%), hypertension (52.2%), COPD (50.5%), angina pectoris (34.4%), and diabetes (24.7%). The overall median survival time for all residents was 24.0 months (IQR: 14.8–29.0), while the median survival time for deceased residents was 15.0 months (IQR: 7.0–21.0), which was 14 months shorter than that of surviving residents.

Table 1 (located at the end of the document) compares characteristics between surviving and deceased residents. Deceased residents had significantly lower left calf circumference (25.0 cm vs. 27.1 cm, *p* = 0.006) and lower serum albumin levels (36.0 g/L vs. 37.0 g/L, *p* = 0.003). BANS-S scores were significantly higher among deceased residents (24.0 vs. 23.0, *p* = 0.024), reflecting poorer functional status. Survivors were more likely to use memantine (24.8% vs. 8.2%, *p* = 0.003) and benzodiazepines (20.8% vs. 8.2%, *p* = 0.023). Nutritional intake status also differed significantly between the groups (*p* = 0.002). Although there was no significant difference in acute medical events within three months before enrollment, high rates of fever and pneumonia were expected among residents with advanced dementia. Additionally, many participants underwent burdensome interventions before enrollment, including oxygen therapy (79.6%), physical restraint (44.6%), antibiotic use (38.7%), ECG monitoring (37.1%), gastric tube placement (31.7%), and urinary catheterization (7.0%), with no significant differences between survivors and deceased residents.

**Table 1:** Baseline characteristics of nursing homes residents with advanced dementia (*n* = 186)

Characteristics	Total sample(*n* = 186)	Survivor(*n* = 101)	Deceased(*n* = 85)	*p*-value
Age in years, median (IQR)	83.5(78.0, 89.0)	83.0(77.0, 88.0)	84.0(78.0, 90.0)	0.215
Female, *n* (%)	112(60.2)	62(61.4)	50(58.8)	0.765
GDS, *n* (%)				0.461
Stage 6	80(43.0)	46(45.5)	34(40)	
Stage 7	106(57.0)	55(54.5)	51(60)	
Duration of survival (months), median (IQR)	24.0(14.8, 29.0)	29.0(26.0, 30.5)	15.0(7.0, 21.0)	< 0.001
BANS-S, median (IQR)	23.0(21.0, 25.3)	23.0(20.0, 25.0)	24.0(22.0, 26.0)	0.024
QUALID, median (IQR)	27.0(23.0, 30.0)	26.0(22.0, 30.0)	27.0(24.5, 30.0)	0.316
Left calf circumference(cm), median (IQR)	26.6(23.0, 29.0)	27.1(23.8, 29.8)	25.0(22.6, 27.6)	0.006
Haemoglobin (g/L), Mean ± SD	120.3 ± 17.2	120.4 ± 17.1	120.2 ± 17.5	0.958
Red blood cell specific Volume (%),median (IQR)	36.9(34.0, 39.0)	36.8(34.0, 39.0)	37.0(34.3, 39.5)	0.896
Albumin (g/L), median (IQR)	37.0(35.0, 40.0)	37.0(35.0, 40.0)	36.0(34.0, 39.0)	0.003
Number of Comorbidities	2.0(1.0, 2.0)	2.0(1.0, 3.0)	2.0(1.0, 2.0)	0.324
Anti-dementia drugs, *n* (%)				
Cholinesterase inhibitors	43(23.1)	27(26.7)	16(18.8)	0.225
Memantine	32(17.2)	25(24.8)	7(8.2)	0.003
Sedative-hypnotic drugs, *n* (%)				
Benzodiazepine hypnotic	28(15.1)	21(20.8)	7(8.2)	0.023
Nonbenzodiazepine hypnotic	4(2.2)	2(2.0)	2(2.4)	0.622
Other sedative-hypnotics	1(0.5)	0(0.0)	1(1.2)	0.457
Anti-psychotic drug, *n* (%)				
Quetiapine	11(5.9)	8(7.9)	3(3.5)	0.232
Olanzapine	14(7.5)	7(6.9)	7(8.2)	0.785
Risperidone	10(5.4)	3(3.0)	7(8.2)	0.190
Other antipsychotics	4(2.2)	1(1.0)	3(3.5)	0.333
Anti-depressant drug, *n* (%)				
Citalopram	2(1.1)	2(2.0)	0(0.0)	0.501
Mirtazapine	2(1.1)	2(2.0)	0(0.0)	0.501
Sertraline hydrochloride	8(4.3)	5(5.0)	3(3.5)	0.729
Fluoxetine hydrochloride	1(0.5)	1(1.0)	0(0.0)	1.000
Nutritional intake status, *n* (%)				0.002
Oral nutritional supplements	15(8.1)	2(2.0)	13(15.3)	
Intravenous nutritional supplements	24(12.9)	16(15.8)	8(9.4)	
Normal diet	147(79.0)	83(82.2)	64(75.3)	
Event within 3 months before enrolled, *n* (%)				
Pneumonia	74(39.8)	39(38.6)	35(41.2)	0.765
Fever	40(21.5)	23(22.8)	17(20)	0.721
UTI	14(7.5)	7(6.9)	7(8.2)	0.737
Stroke	7(3.8)	6(5.9)	1(1.2)	0.128
Pressure ulcer	7(3.8)	4(4.0)	3(3.5)	1.000
Fracture	4(2.2)	3(3.0)	1(1.2)	0.627
Interventions used within 3 months before enrolled, *n* (%)				
Oxygen therapy	148 (79.6)	81 (80.2)	67 (78.8)	0.856
Physical restraint	83 (44.6)	52 (51.5)	31 (36.5)	0.054
Antibiotics use	72 (38.7)	42 (41.6)	30 (35.3)	0.450
ECG monitoring	69 (37.1)	42 (41.6)	27 (31.8)	0.175
Gastric tube placement	59 (31.7)	30 (29.7)	29 (34.1)	0.531
Urinary catheterization	13 (7.0)	5 (5.0)	8 (9.4)	0.261

*IQR* interquartile range, *SD *standard deviation

Univariate and multivariable Cox proportional hazards regression analyses were conducted, adjusting for age, gender, and baseline variables with significant group differences (see Table 2 at the end of the document). Schoenfeld residuals were used to validate the proportional hazards assumption. The global test yielded χ² = 12.88 with 9 degrees of freedom (*p* = 0.168), and all covariate-specific *p*-values exceeded 0.05 (range: 0.078–0.887), as detailed in Table S4 (Additional File 1). Graphical inspection further confirmed that Schoenfeld residuals were randomly distributed around zero (see Fig. S1 in Additional File 1). These results indicate that all covariates met the proportional hazards assumption and were included in the final model. In univariate analysis, higher BANS-S scores and oral nutritional supplement use were associated with increased mortality, while memantine use, greater left calf circumference, and higher albumin levels were protective factors. In the multivariable model adjusting for all variables, oral nutritional supplement use remained a significant predictor of mortality (AHR: 2.93, 95% CI: 1.530–5.611), whereas memantine use (AHR: 0.438, 95% CI: 0.197–0.975) and higher albumin levels (HR: 0.927, 95% CI: 0.871–0.987) were independently associated with improved survival. In the final multivariable regression model, memantine use, nutritional intake status, and albumin levels were retained as significant factors. Adjusted HRs (95% CIs) for mortality were 0.416 (0.190, 0.907) for memantine use, 0.914 (0.861, 0.970) for higher albumin levels, and 3.006 (1.593, 5.671) for oral nutritional supplement use.

**Table 2:** Cox regression analysis of prognostic factors associated with mortality for residents with advanced dementia

Variables	Univariate Cox regression analysis			Multivariable Cox regression analysis			Multivariable Cox regression analysis		
	HR	95% CI	*p*-value	Adjusted HR	95% CI	*p*-value	Adjusted HR	95% CI	*p*-value
Age	1.017	0.99–1.044	0.223	1.006	0.977–1.036	0.684			
Male	1.305	0.847–2.012	0.227	1.466	0.932–2.307	0.098			
BANS-S	1.081	1.013–1.154	0.018	1.027	0.950–1.111	0.501			
Using Memantine	0.361	0.167–0.784	0.01	0.438	0.197–0.975	0.043	0.416	0.190–0.907	0.028
Using Benzodiazepine hypnotic	0.477	0.22–1.034	0.061	0.696	0.314–1.543	0.372			
Left calf circumference	0.94	0.889–0.995	0.032	0.989	0.923–1.059	0.741			
Albumin	0.904	0.854–0.957	0.001	0.927	0.871–0.987	0.018	0.914	0.861–0.970	0.003
Nutritional intake status									
Normal diet (Reference)	-	-	-	-	-	-	-	-	-
Oral nutritional supplements	4.369	2.386-8.000	< 0.001	2.93	1.530–5.611	0.001	3.006	1.593–5.671	0.001
Intravenous nutritional supplements	0.741	0.355–1.546	0.425	0.628	0.290–1.361	0.238	0.63	0.295–1.344	0.232

*HR* hazard ratio, *CI *confidence interval

**Fig. 1:**
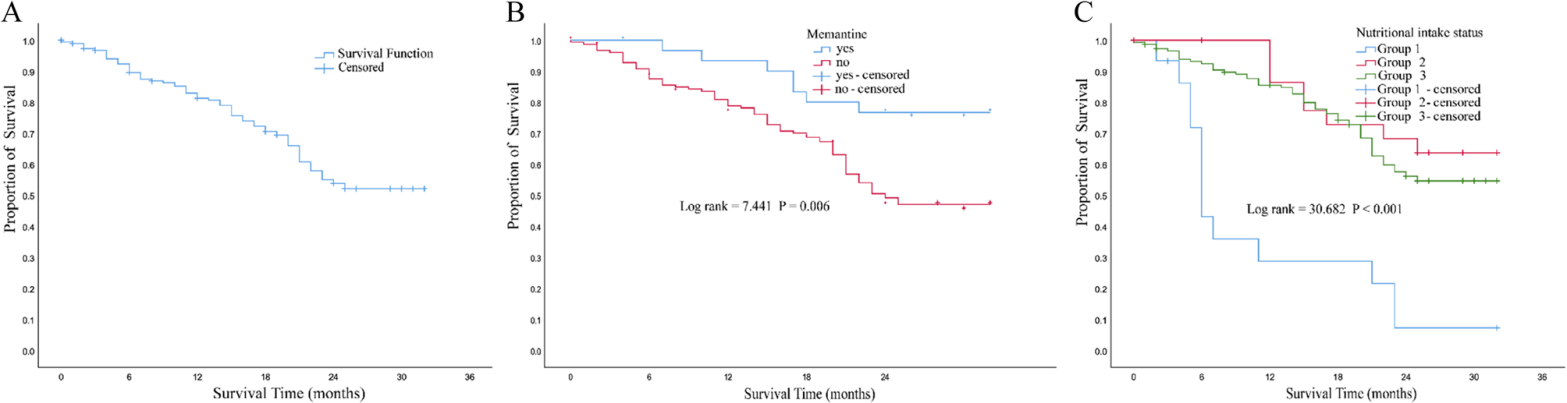
Survival over a 32-month follow-up period for residents with advanced dementia. **A** Kaplan-Meier survival analyses of all residents affected by advanced dementia. **B** Kaplan-Meier survival analyses for residents affected by advanced dementia who were or were not memantine users. **C** Kaplan-Meier survival analyses for residents with advanced dementia with differing nutritional intake status

Survival curves derived with the Kaplan-Meier method and corresponding survival rates are summarized in Fig. 1; Table 3 (located at the end of the document). The overall rates at 6 months, 1 year, and 2 years were 89.6%, 81.2%, and 53.8%, respectively, with an overall survival rate of 52.0%. Participants using memantine had significantly higher survival rates at all time points compared to non-users (100%, 93.3%, 76.7%, and 76.7% vs. 87.5%, 78.8%, 49.0%, and 46.9%; *p* = 0.006). In comparison, residents receiving oral nutritional supplements had significantly lower survival rates than those on a regular diet (6-month: 43.1%, 1-year: 28.7%, 2-year: 7.2%, overall: 7.2% vs. 92.4%, 85.5%, 56.0%, 54.5%; *p* < 0.001). Additionally, the survival rates of participants receiving intravenous nutritional supplements showed no statistically significant difference compared to those on a regular diet (6-month: 100%, 1-year: 86.4%, 2-year: 68.2%, overall: 63.6% vs. 92.4%, 85.5%, 56.0%, 54.5%; *p* = 0.424), but were higher than those receiving ONS.

**Table 3:** Survival rate of different variables during follow-up for 186 residents with advanced dementia

Variables	6-month survival rate	1-year survival rate	2-year survival rate	Overall survival rate
Overall study population	89.6%	81.2%	53.8%	52.0%
Memantine				
No	87.50%	78.80%	49.00%	46.90%
Yes	100%	93.30%	76.70%	76.70%
Nutritional intake status				
Normal diet	92.4%	85.5%	56.0%	54.5%
Oral nutritional supplements	43.10%	28.70%	7.20%	7.20%
Intravenous nutritional supplements	100%	86.4%	68.2%	63.6%

### Sensitivity analyses

A sensitivity analysis was conducted to exclude residents lost to follow-up, and the results remained consistent with the primary analysis. Compared to deceased residents, surviving residents exhibited greater left calf circumference and higher albumin concentrations and were more likely to have used memantine and benzodiazepines. Nutritional intake status continued to show significant differences between groups (see Table S5 in Additional File 1). In the multivariable regression analysis, memantine use and higher albumin levels were linked to lower mortality risk, while oral nutritional supplementation remained a significant risk factor for death (see Table S6 in Additional File 1). Additionally, survival rates were compared across different groups. Residents receiving oral nutritional supplements exhibited significantly lower 6-month, 1-year, 2-year, and overall survival rates compared to those on a normal diet; however, no statistically significant difference was observed in survival rates between recipients of intravenous nutritional supplements and those on a normal diet. Similarly, residents using memantine exhibited significantly higher survival rates than those not taking the medication (Table S7 and Fig. S2 in Additional File 1). These findings further reinforced the trends observed in the categorical analysis.

## Discussion

This study followed 186 nursing home residents with advanced dementia over a 32-month period. Measured rates of 6-month, 1-year, 2-year, and overall survival were 89.6%, 81.2%, 53.8%, and 52.0%, respectively. Multivariable analysis identified memantine use as a protective factor for survival among residents with advanced dementia, whereas oral nutritional supplementation was independently associated with increased mortality. Notably, mortality rates at all time points were significantly lower among residents using memantine compared to those not taking the medication. To the best of our knowledge, this study is the first to suggest that memantine use may reduce mortality among nursing home residents with advanced dementia.

The 6-month survival rate observed in this study (89.6%) was significantly higher than that observed by Mitchell SL et al. (75.3%) [5]. Still, it was largely consistent with results from a prospective cohort study conducted in Chongqing, China (95.7%) [14]. One possible explanation for these discrepancies is the variation in inclusion criteria. The present study, like the Chongqing study, included participants of GDS stage 6 or higher, whereas the other study limited inclusion to individuals at GDS stage 7, indicating more severe functional impairments. Prior research has established an association between greater functional decline and lower survival rates [24, 25]. Additionally, cultural differences in end-of-life care approaches may contribute to differences in survival. In China, the limited availability of palliative care education, coupled with deep-rooted cultural beliefs emphasizing longevity, often leads families to pursue aggressive medical interventions [26]. The Confucian principle that “filial piety is the foundation of all virtues” places considerable societal and psychological pressure on children, who may fear that discontinuing active treatment for their parents could be perceived as neglectful [26–28]. This underscores the need for improved resources and strategies to support family members in making informed decisions and for nursing homes to enhance staff training in end-of-life care for residents with advanced dementia.

As far as we are aware, this study represents the first comprehensive investigation of the association between medication profiles, including anti-dementia agents, sedative-hypnotics, anti-psychotics, and anti-depressants, and mortality among nursing home residents with advanced dementia. These results identify memantine as a novel prognostic marker associated with improved survival in the study population. Memantine has received approval for treating moderate to severe dementia for nearly two decades [29, 30]. However, its efficacy in advanced dementia remains a topic of debate. Shega et al. reported that a subset of hospice care medical directors observed clinical benefits associated with continued memantine use in advanced dementia [31]. Conversely, a systematic review suggested that the benefits of memantine for moderate to severe dementia were minimal [32]. Some studies have indicated that oral memantine may improve functional outcomes in patients with advanced dementia [12], while research by Calvo-Perxas et al. demonstrated that memantine is commonly prescribed for elderly individuals with severe Alzheimer’s disease [33]. To date, no studies have explicitly examined whether memantine use influences survival in advanced dementia. Our study showed that memantine use is associated with prolonged survival in nursing home residents with advanced dementia. These results indicate that continued memantine treatment may be beneficial in extending the survival period of these residents.

Research on the impact of ONS in advanced dementia remains limited. A systematic review by Randi et al. demonstrated that ONS interventions increased energy and protein intake, thereby improving nutritional status in dementia patients. However, the study did not assess survival or functional outcomes [34]. The present study found that residents receiving ONS had increased mortality. A recent cross-sectional pilot study examined decision-making pertaining to ONS in advanced dementia, revealing that physicians, dietitians, and surrogate decision-makers held differing opinions on whether ONS could prolong life. Most dietitians agree that ONS does not extend lifespan [35]. Residents receiving ONS may have underlying conditions such as inadequate food intake or severe malnutrition. In the present study, lower albumin levels were associated with higher mortality, aligning with previous research demonstrating that poor nutritional status significantly increases the risk of death in dementia patients [5, 13, 36, 37]. This may partly explain the increased mortality associated with ONS. Another potential factor contributing to increased mortality is the risk of aspiration. Dysphagia affects up to 86% of individuals with advanced dementia, making it one of the most common functional impairments in this population. It substantially elevates the risk of aspiration pneumonia, a major contributor to mortality in these residents [38]. The use of oral nutritional supplements (ONS) may lead to aspiration in this population, particularly among those with impaired swallowing function, thereby increasing the risk of aspiration pneumonia and potentially contributing to mortality [39, 40]. Furthermore, residents receiving intravenous nutritional supplements are not exposed to the risk of aspiration, which may partially account for their higher survival rates than those receiving oral nutritional supplements.Given these risks, the decision to initiate ONS in patients with advanced dementia should be carefully considered through thorough discussions between healthcare providers and caregivers.

Several limitations of this study warrant consideration. First, the sample population was predominantly female, potentially underestimating male-associated mortality, as some studies suggest that men face a higher risk of death in dementia [41, 42]. Second, no differentiation was performed between specific subtypes of advanced dementia, despite evidence indicating that survival varies by dementia subtype [43]. As a result, these findings may not be generalizable to all forms of advanced dementia. Third, potential interactions between predictors may not have been detected due to the limited sample size. Fourth, the small sample size (*n* = 186) may have reduced statistical power and increased the risk of Type II error. Fifth, all participants were recruited from a single geographic region, Chengdu, China, where specific population characteristics, healthcare infrastructure, and environmental factors may influence outcomes, thus limiting the generalizability of the results. Sixth, the absence of data on other health-related factors, such as systemic inflammatory markers and caregiver quality, may have introduced residual confounding, potentially affecting the causal interpretation of the observed associations with survival. Finally, while this study focused on survival outcomes, quality of life is an equally important consideration that warrants further investigation.

## Conclusions

This prospective cohort study revealed that memantine use is a protective factor for survival among nursing home residents exhibiting advanced dementia in southwestern China. Over a 32-month follow-up period, the overall survival rate was 52.0%. These findings serve as a source of valuable insights into the prognosis of advanced dementia patients and highlight the need for optimized resource allocation in end-of-life care.

## Supplementary Information

Additional File 1: Table S1. Global deterioration scale (GDS) clinical characteristics by stage. Table S2. Scoring guidelines for the quality of life in late-stage dementia (QUALID) scale. Table S3. Scoring guidelines for the bedford alzheimer nursing severity-subscale (BANS-S). Table S4. Schoenfeld residuals test for proportional hazards assumption. Table S5. Baseline characteristics of nursing homes residents with advanced dementia. Table S6. Cox regression analysis of prognostic factors associated with mortality for residents with advanced dementia. Table S7. Survival rate of different variables during follow-up for 174 residents with advanced dementia. Figure S1. Plots of Schoenfeld residuals against time. Figure S2. Survival over a 32-month follow-up period was analyzed in residents with advanced dementia, excluding those lost to follow-up.

## Data Availability

The datasets used and/or analysed during the current study are available from the corresponding author on reasonable request.

## Abbreviations

GBD: Global Burden of Disease
DALYs: Disability-adjusted life-years
ADOD: Alzheimer's disease and other dementias
GDS: The global deterioration scale
CASCADE: The choices, attitudes, and strategies for care of advanced dementia at the end-of-life study
ADEPT: Advanced Dementia Prognostic Tool
IADL: Instrumental activities of daily living
ADL: Activities of daily living
QUALID: Quality of Life in Late-Stage Dementia
BANS-S: Bedford Alzheimer Nursing Severity Scale
ONS: Oral nutritional supplements
COPD: Chronic obstructive pulmonary disease
UTI: Urinary tract infections
ECG: Electrocardiogram
SD: Standard deviation
IQR: Interquartile range
HR: Hazard ratio
CI: Confidence intervals
