# Supplementary material for: Prognostic predictors of mortality among individuals with advanced dementia residing in nursing homes: a prospective cohort study
Source: BMC Geriatr. 2025 Oct 22;25:796. doi: 10.1186/s12877-025-06350-5 (PMC12542335; doi:10.1186/s12877-025-06350-5)
Supplement: Supplementary file 1 — Additional File 1: Table S1. Global deterioration scale (GDS) clinical characteristics by stage. Table S2. Scoring guidelines for the quality of life in late-stage dementia (QUALID) scale. Table S3. Scoring guidelines for the bedford alzheimer nursing severity-subscale (BANS-S). Table S4. Schoenfeld residuals test for proportional hazards assumption. Table S5. Baseline characteristics of nursing homes residents with advanced dementia. Table S6. Cox regression analysis of prognostic factors associated with mortality for residents with advanced dementia. Table S7. Survival rate of different variables during follow-up for 174 residents with advanced dementia. Figure S1. Plots of Schoenfeld residuals against time. Figure S2. Survival over a 32-month follow-up period was analyzed in residents with advanced dementia, excluding those lost to follow-up. [file 12877_2025_6350_MOESM1_ESM.docx]

**Table S1.** **Global Deterioration Scale (GDS) Clinical Characteristics by Stage**

| **Stage 1: No Cognitive Decline**  **Clinical characteristics:** Patients in stage l appear normal clinically; they have no complaints of memory defcit,and a clinical interview does not elicit evidence of memory defcit. |
| --- |
| **Stage 2: Very Mild Cognitive Decline**  **Clinical characteristics:**This is the phase of forget-fulness. Self-complaints of poor memory typically manifest in the following ways: (1) Forgetting the location of familiar items; (2) Forgetting the names of acquaintances, although clinical examination reveals no objective evidence of memory impairment. No objective functional impairment is observed in occupational or social settings, and concern about these symptoms is appropriate. |
| **Stage 3: Mild Cognitive Decline**  **Clinical characteristics:** The earliest clear cognitive deficits, with two or more of the following manifestations: (1) The patient becomes disoriented in unfamiliar places; (2) Colleagues notice a relative decline in the patient's work ability; (3) Family members observe difficulty in recalling words or names; (4) Retaining little information after reading an article or book; (5) Reduced ability to remember newly learned names; (6) May misplace or lose valuables; (7) Clinical examination reveals evidence of diminished attention. Objective evidence of memory decline may only be obtained through comprehensive assessment. Mild impairment in occupational and social functioning may be present. Patients begin to exhibit denial accompanied by mild to moderate anxiety symptoms. |
| **Stage 4:Moderate Cognitive Decline**  **Clinical characteristics：**Marked cognitive deficits manifest in the following aspects: (1) Reduced knowledge of current and recent events; (2) Impaired memory of personal history; (3) Impaired concentration demonstrated by serial subtraction testing; (4) Declined ability to perform tasks such as traveling and managing finances. However, the following three domains typically remain intact: (1) Orientation to time and person; (2) Recognition of familiar individuals and faces; (3) Ability to navigate familiar places. Patients cannot complete complex tasks, exhibit prominent denial as a psychological defense mechanism, display blunted affect, and avoid challenging situations. |
| **Stage 5: Moderately Severe Cognitive Decline**  **Clinical characteristics:** Patients require assistance with daily living; during examination, they cannot recall highly familiar personal details after prolonged effort, such as their address, long-used telephone number, names of close relatives (e.g., grandchildren), the names of their high school or university, or demonstrate topographical disorientation. Educated individuals show difficulty performing serial subtractions (e.g., 40 minus 4 serially or 20 minus 2 serially). At this stage, patients retain knowledge of significant personal and familial events. They know their own name and typically recall their spouse's and only child's name. While able to eat and maintain continence without assistance, many cannot select appropriate clothing. |
| **Stage 6: Severe Cognitive Decline**  **Clinical characteristics:** Inability to recall the spouse's name and substantial forgetting of recent experiences/events. Fragmentary knowledge of past experiences persists but is severely limited. Patients typically lose environmental awareness (disorientation to time/year/season) and may struggle with basic arithmetic (<10). Requiring full daily living assistance, they may experience incontinence and need accompaniment outdoors, occasionally reaching familiar locations. Circadian rhythm disruption occurs. Self-identification remains largely intact, with frequent ability to distinguish familiar/unfamiliar persons. Unstable personality and emotional changes emerge, including: (1) Delusional behaviors (e.g., accusing spouses of infidelity, conversing with imaginary figures or mirror images); (2) Compulsive symptoms (e.g., repetitive cleaning rituals); (3) Anxiety/agitation with previously uncharacteristic violent behaviors; (4) Cognitive abulia (loss of volitional capacity due to inability to sustain thoughts for behavioral decisions). |
| **Stage 7: Very Severe Cognitive Decline**  **Clinical characteristics:** Patients exhibit complete loss of verbal function, typically reduced to unintelligible vocalizations. Urinary incontinence is present, requiring assistance for feeding and toileting. Fundamental psychomotor skills are abolished (e.g., inability to walk), demonstrating apparent loss of cortical control over motor function. Diffuse cortical neurological signs and symptoms are commonly observed. |

Reisberg B, Ferris SH, de Leon MJ, Crook T: The Global Deterioration Scale for assessment of primary degenerative dementia. *Am J Psychiatry* 1982, 139(9):1136-1139.

**Table S2. Scoring Guidelines for the Quality of Life in Late-Stage Dementia (QUALID) Scale**

| **Which response best describes _ over the past week…** | |
| --- | --- |
| **A** | [S] smiles 1. spontaneously once or more each day 2. spontaneously less than once each day 3. only in response to external stimuli; at least once each day 4. only in response to external stimuli; less than once each day 5. rarely, if at all |
| **B** | [S] appears sad 1. rarely or never 2. only in response to external stimuli; less than once each day 3. only in response to external stimuli; at least once each day 4. for no apparent reason less than once each day 5. for no apparent reason once or more each day |
| **C** | [S] cries 1. rarely or never 2. only in response to external stimuli; less than once each day 3. only in response to external stimuli; at least once each day 4. for no apparent reason less than once each day 5. for no apparent reason once each day or more |
| **D** | [S] has a facial expression of discomfort - appears unhappy or in pain (looks worried, grimaces, furrowed or turned down brow) 1. rarely or never 2. less than once each day 3. at least once each day 4. nearly half of each day 5. most of each day |
| **E** | [S] appears physically uncomfortable –he/she squirms, writhes, frequently changes position 1. rarely or never 2. less than once each day 3. at least once each day 4. nearly half of each day 5. most of each day |
| **F** | [S] makes statements or sounds that suggest discontent, unhappiness or discomfort (complains, groans, screams) 1. rarely or never 2. only in response to external stimuli; less than once each day 3. only in response to external stimuli; at least once each day 4. without cause less than once each day 5. without cause once or more each day |
| **G** | [S] is irritable or aggressive (becomes angry, curses, pushes or attempts to hurt others) 1. rarely or never 2. only in response to external stimuli; less than once each day 3. only in response to external stimuli; at least once each day 4. without cause less than once each day 5. without cause once or more each day |
| **H** | [S] enjoys eating 1. at most meals and snacks 2. twice a day 3. at least once a day 4. less than once each day 5. rarely or never |
| **I** | [S] enjoys touching/being touched 1. almost always; almost always initiates touching 2. more than half the time; sometimes initiates touching 3. half the time; never initiates touching, but doesn't resist touching/being 4. less than half the time; often or frequently resists touching/being touched 5. rarely or never; almost always resists touching/being touched |
| **J** | [S] enjoys interacting or being with others 1. almost always; almost always initiates interaction with others 2. more than half the time; sometimes initiates interaction with others 3. half the time; never initiates interaction, but doesn't resist interaction with others 4. less than half the time; often or frequently resists interacting with others 5. rarely or never; almost always resists interacting with others |
| **K** | [S] appears emotionally calm and comfortable 1. most of each day 2. more than half of each day 3. half of each day 4. less than half of each day 5. rarely or never |
| **Total Score:** | *(Sum of all item scores, ranging from 11 to 55. Higher scores indicate better quality of life.)* |

Weiner MF, Martin-Cook K, Svetlik DA, Saine K, Foster B, Fontaine CS: The quality of life in late-stage dementia (QUALID) scale. *J Am Med Dir Assoc* 2000, 1(3):114-116.

**Table S3**. **Scoring Guidelines for the Bedford Alzheimer Nursing Severity-Subscale (BANS-S)**

| **Assessment Item** | **Grading Criteria** | **Score** |
| --- | --- | --- |
| **Dressing** |  |  |
|  | [1] Usually independent | 1 |
|  | [2] Requires minimal assistance | 2 |
|  | [3] Requires moderate assistance but is not totally dependent | 3 |
|  | [4] Completely dependent | 4 |
| **Sleep–wake cycle disturbance** |  |  |
|  | [1] Usually regular sleep-wake cycle | 1 |
|  | [2] Sometimes irregular | 2 |
|  | [3] Frequently irregular | 3 |
|  | [4] Severely disrupted sleep-wake cycle | 4 |
| **Speech** |  |  |
|  | [1] Preserved | 1 |
|  | [2] Mild inability to speak | 2 |
|  | [3] Occasional inability | 3 |
|  | [4] Complete aphasia | 4 |
| **Eating** |  |  |
|  | [1] Independent | 1 |
|  | [2] Requires minimal assistance and/or prompting | 2 |
|  | [3] Requires moderate assistance and/or prompting | 3 |
|  | [4] Completely dependent | 4 |
| **Ambulation** |  |  |
|  | [1] Always ambulates independently | 1 |
|  | [2] Sometimes ambulates independently | 2 |
|  | [3] Ambulates only with assistance | 3 |
|  | [4] Unable to ambulate even with assistance | 4 |
| **Muscle rigidity/contractions** |  |  |
|  | [1] Very supple with fully preserved joint mobility | 1 |
|  | [2] Partially supple with mild impairment of joint mobility | 2 |
|  | [3] Partially rigid | 3 |
|  | [4] Contracted | 4 |
| **Eye Contact** |  |  |
|  | [1] Maintained | 1 |
|  | [2] Usually maintained | 2 |
|  | [3] Rarely maintained | 3 |
|  | [4] Completely absent | 4 |
| **Total Score:** | (Sum of all item scores, ranging from 7 to 28. Higher scores indicate poorer function.) | |

Volicer L, Hurley AC, Lathi DC, Kowall NW: Measurement of severity in advanced Alzheimer's disease. *J Gerontol* 1994, 49(5):M223-226.

**Table S4. Schoenfeld Residuals Test for Proportional Hazards Assumption**

| **Covariate** | **χ²** | **df** | **p-value** |
| --- | --- | --- | --- |
| Age | 0.020 | 1 | 0.887 |
| Sex | 1.917 | 1 | 0.166 |
| BANS-S | 3.112 | 1 | 0.078 |
| Memantine | 0.077 | 1 | 0.781 |
| Benzodiazepine | 1.362 | 1 | 0.243 |
| Left calf circumference | 2.330 | 1 | 0.127 |
| Albumin | 2.519 | 1 | 0.112 |
| Nutritional intake status | 0.830 | 2 | 0.660 |
| **Global test** | **12.878** | **9** | **0.168** |

**Table S****5**. **Baseline characteristics of nursing homes residents with advanced dementia (n = 174)**

| **Characteristics** | **Total sample（n=174）** | **Survivor（n=89）** | **Deceased（n=85）** | ***P*-value** |
| --- | --- | --- | --- | --- |
| Age in years, median (IQR) | 83.5（78.0，89.0） | 83.0（77.0，88.0） | 84.0（78.0，90.0） | 0.256 |
| Female, n (%) | 109（62.6） | 59（66.3） | 50（58.8） | 0.348 |
| GDS, n (%) |  |  |  |  |
| Stage 6 | 70（40.2） | 36（40.4） | 34（40） | 1.000 |
| Stage 7 | 104（59.8） | 53（59.6） | 51（60） |  |
| Duration of survival (months), median (IQR) | 25.0（15.0，29.3） | 29.0（29.0，31.0） | 15.0（7.0，21.0） | <0.001 |
| BANS-S, median (IQR) | 24.0（21.0，26.0） | 23.0（20.5，25.0） | 24.0（22.0，26.0） | 0.086 |
| QUALID, median (IQR) | 27.0（24.0，30.0） | 27.0（23.0，30.0） | 27.0（24.5，30.0） | 0.556 |
| Left calf circumference(cm), median (IQR) | 26.1（23.0，28.8） | 27.0（23.5，29.0） | 25.0（22.6，27.6） | 0.046 |
| Haemoglobin (g/L), Mean±SD | 120.3±17.3 | 120.4±17.2 | 120.2±17.5 | 0.949 |
| Red blood cell specific Volume (%),median (IQR) | 36.7±4.5 | 36.7±4.3 | 36.6±4.6 | 0.870 |
| Albumin (g/L), median (IQR) | 37.0（35.0，40.0） | 38.0（35.0，40.0） | 36.0（34.0，39.0） | 0.002 |
| Number of Comorbidities | 2.0（1.0，2.0） | 2.0（1.0，2.0） | 2.0（1.0，2.0） | 0.428 |
| Anti-dementia drugs, n (%) |  |  |  |  |
| Cholinesterase inhibitors | 40（23.0） | 24（27.0） | 16（18.8） | 0.213 |
| Memantine | 29（16.7） | 22（24.7） | 7（8.2） | 0.004 |
| Sedative-hypnotic drugs, n (%) |  |  |  |  |
| Benzodiazepine hypnotic | 25（14.4） | 18（20.2） | 7（8.2） | 0.030 |
| Nonbenzodiazepine hypnotic | 4（2.3） | 2（2.2） | 2（2.4） | 1.000 |
| Other sedative-hypnotics | 1（0.6） | 0（0.0） | 1（1.2） | 0.489 |
| Anti-psychotic drug, n (%) |  |  |  |  |
| Quetiapine | 9（5.2） | 6（6.7） | 3（3.5） | 0.497 |
| Olanzapine | 13（7.5） | 6（6.7） | 7（8.2） | 0.778 |
| Risperidone | 9（5.2） | 2（2.2） | 7（8.2） | 0.094 |
| Other antipsychotics | 4（2.3） | 1（1.1） | 3（3.5） | 0.359 |
| Anti-depressant drug, n (%) |  |  |  |  |
| Citalopram | 2（1.1） | 2（2.2） | 0（0.0） | 0.497 |
| Mirtazapine | 2（1.1） | 2（2.2） | 0（0.0） | 0.497 |
| Sertraline hydrochloride | 7（4.0） | 4（4.5） | 3（3.5） | 1.000 |
| Fluoxetine hydrochloride | 1（0.6） | 1（1.1） | 0（0.0） | 1.000 |
| Nutritional intake status, n (%) |  |  |  |  |
| Oral nutritional supplements | 14（8.0） | 1（1.1） | 13（15.3） | 0.001 |
| Intravenous nutritional supplements | 22（12.6） | 14（15.7） | 8（9.4） |  |
| Normal diet | 138（79.3） | 74（83.1） | 64（75.3） |  |
| Event within 3 months before enrolled, n (%) |  |  |  |  |
| Pneumonia | 67（38.5） | 32（36.0） | 35（41.2） | 0.534 |
| Fever | 34（19.5） | 17（19.1） | 17（20） | 1.000 |
| UTI | 14（8.0） | 7（7.9） | 7（8.2） | 1.000 |
| Stroke | 7（4.0） | 6（6.7） | 1（1.2） | 0.118 |
| Pressure ulcer | 5（2.9） | 2（2.2） | 3（3.5） | 0.677 |
| Fracture | 2（1.1） | 1（1.1） | 1（1.2） | 1.000 |
| Interventions used within 3 months before enrolled, n (%) |  |  |  |  |
| Oxygen therapy | 141(81.0) | 74(83.1) | 67(78.8) | 0.563 |
| Physical restraint | 79 (44.6) | 48 (53.9) | 31 (36.5) | 0.051 |
| Antibiotics use | 67(38.5) | 37(41.6) | 30(35.3) | 0.438 |
| Ecg monitoring | 67(38.5) | 40(44.9) | 27(31.8) | 0.087 |
| Gastric tube placement | 56(32.2) | 27(30.3) | 29(34.1) | 0.629 |
| Urinary catheterization | 13(7.5) | 5(5.6) | 8(9.4) | 0.397 |

Note: IQR = interquartile range, SD = standard deviation

**Table S****6. Cox regression analysis of prognostic factors associated with mortality for** **residents with** **advanced dementia**

| **Variables** | **Univariate Cox regression analysis** | | |  | **Multivariable Cox regression analysisa** | | |  | **Multivariable Cox regression analysisa** | | |
| --- | --- | --- | --- | --- | --- | --- | --- | --- | --- | --- | --- |
|  | HR | 95% CI | p-value |  | Adjusted HR | 95% CI | p-value |  | Adjusted HR | 95% CI | p-value |
| Age | 1.015 | 0.988-1.042 | 0.274 |  | 1.003 | 0.975-1.032 | 0.84 |  |  |  |  |
| Male | 1.354 | 0.879-2.087 | 0.169 |  | 1.48 | 0.945-2.318 | 0.087 |  |  |  |  |
| Using Memantine | 0.368 | 0.170-0.798 | 0.011 |  | 0.431 | 0.195-0.951 | 0.037 |  | 0.429 | 0.197-0.938 | 0.034 |
| Using Benzodiazepine hypnotic | 0.473 | 0.218-1.025 | 0.058 |  | 0.694 | 0.314-1.535 | 0.367 |  |  |  |  |
| Left calf circumference | 0.946 | 0.893-1.001 | 0.053 |  | 0.986 | 0.926-1.050 | 0.671 |  |  |  |  |
| Albumin | 0.901 | 0.851-0.955 | <0.001 |  | 0.917 | 0.861-0.977 | 0.008 |  | 0.911 | 0.857-0.968 | 0.003 |
| Nutritional intake status |  |  |  |  |  |  |  |  |  |  |  |
| Normal diet (Reference) | - | - | - |  | - | - | - |  | - | - | - |
| Oral nutritional supplements | 4.247 | 2.319-7.777 | <0.001 |  | 2.842 | 1.483-5.447 | 0.002 |  | 2.873 | 1.518-5.437 | 0.001 |
| Intravenous nutritional supplements | 0.725 | 0.348-1.513 | 0.392 |  | 0.588 | 0.274-1.265 | 0.174 |  | 0.605 | 0.283-1.295 | 0.196 |

**Note:** HR: hazard ratio; CI: confidence interval.

**Table S7.** **Survival rate of different variables during follow-up for 174** **residents with advanced dementia**

| **Variables** | **6-month survival rate** | **1-year survival rate** | **2-year survival rate** | **Overall survival rate** |
| --- | --- | --- | --- | --- |
| Overall study population | 89.1% | 80.5% | 52.9% | 51.1% |
| Memantine |  |  |  |  |
| No | 86.9% | 77.9% | 48.3% | 46.2% |
| Yes | 100% | 93.1% | 75.9% | 75.9% |
| Nutritional intake status |  |  |  |  |
| Normal diet | 92.0% | 84.8% | 55.1% | 53.6% |
| Oral nutritional supplements | 42.9% | 28.6% | 7.1% | 7.1% |
| Intravenous nutritional supplements | 100% | 86.4% | 68.2% | 63.6% |

**Fig S1. Plots of Schoenfeld residuals against time**


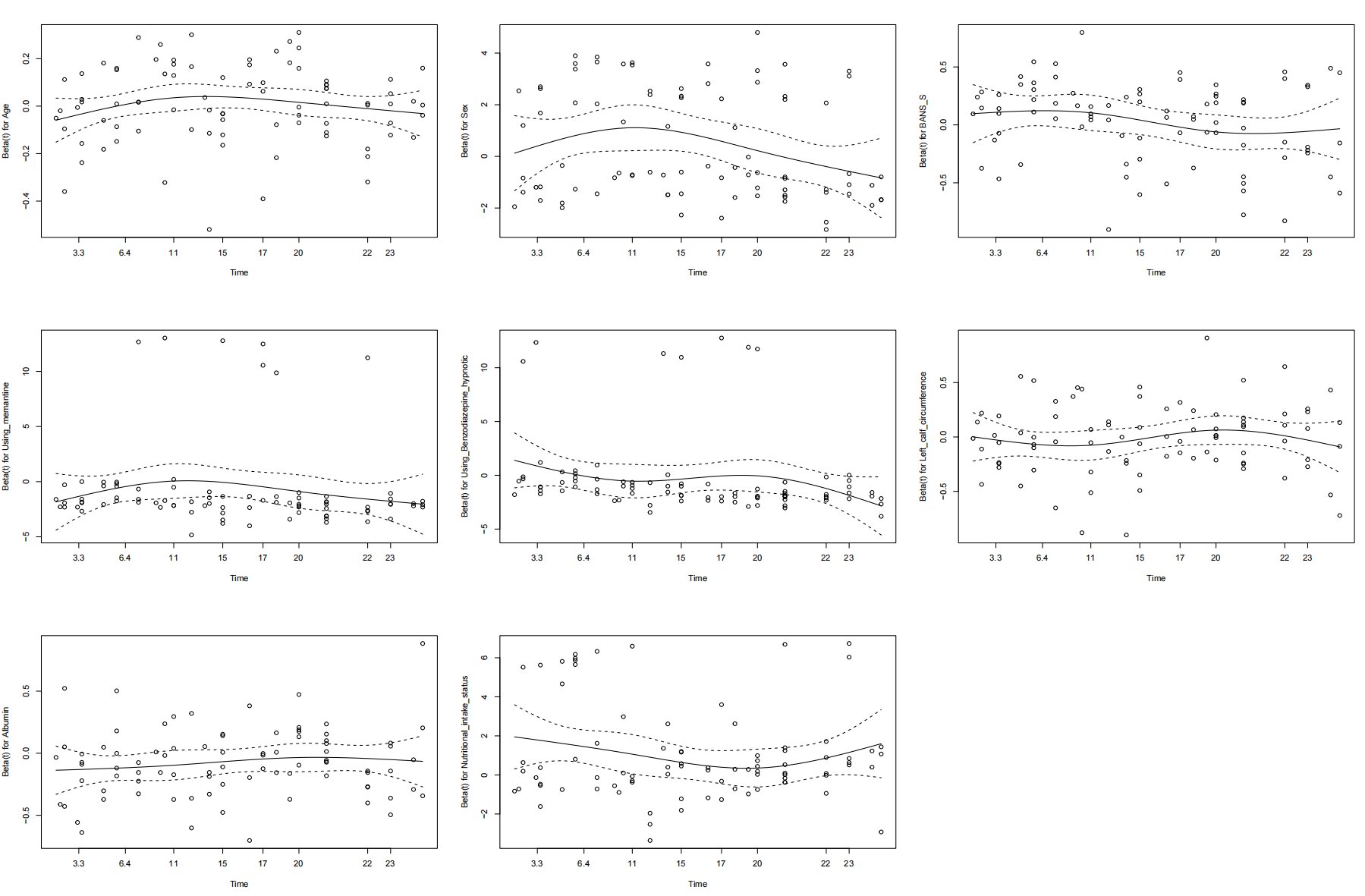


**Figure S2**


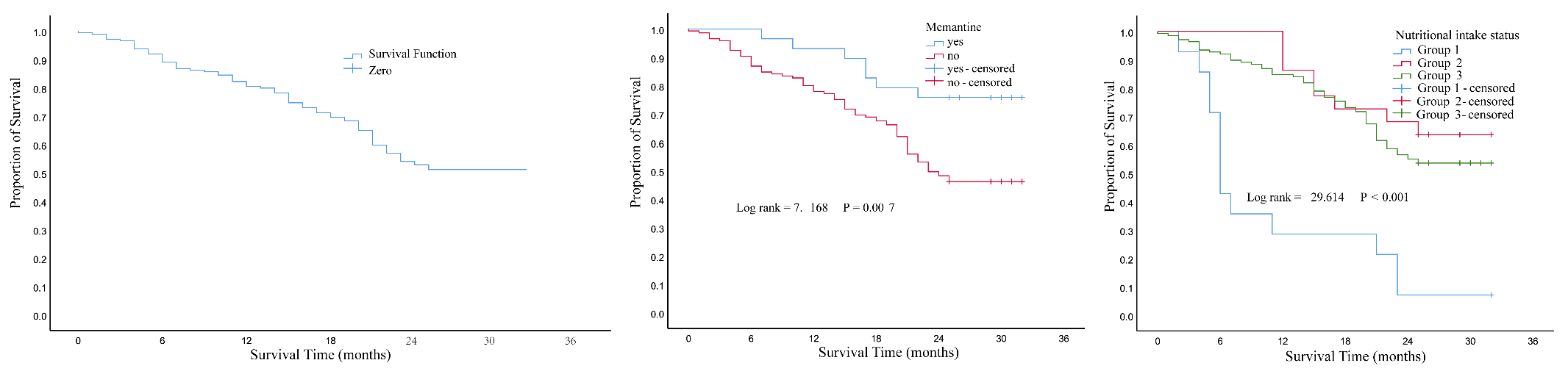


**Figure S2：**Survival over a 32-month follow-up period was analyzed in residents with advanced dementia, excluding those lost to follow-up. (A) Kaplan-Meier survival analyses of all residents affected by advanced dementia. (B) Kaplan-Meier survival analyses for residents affected by advanced dementia who were or were not memantine users. (C) Kaplan-Meier survival analyses for residents with advanced dementia with differing nutritional intake status.
